# Molecular Cloning, Expression and Characterization of Para Flagellar Rod Protein 1 of *Trypanosoma evansi*

**Published:** 2018

**Authors:** Biswa RANJAN MAHARANA, Anup KUMAR TEWARI, Naduvanahalli RAJANNA SUDHAKAR, Chinmoy MISHRA

**Affiliations:** 1. Dept. of Parasitology, Referral Veterinary Diagnostic & Extension Center, LUVAS, Haryana, India; 2. Division of Parasitology, Indian Veterinary Research Institute, Izatnagar, U.P., India; 3. Division of Animal Genetics and Breeding, OUAT, Odisha, India

**Keywords:** Para flagellar rod protein 1, SDS-PAGE, *Trypanosoma evansi*, Western blot

## Abstract

**Background:**

Antigenic variation allows the trypanosomes to evade the potentially destructive host immune response and is an important reason for failure to develop a protective vaccine. Among the non-variant structural proteins, paraflagellar rod protein (PFR) is a prospective vaccine target owing to its role in the active movement of the parasite.

**Methods:**

The PFR1 gene was cloned in pET-32a expression vector and after confirmation by restriction digestion, expressed as a Histidine-tagged fusion protein, in BL21 DE3 strain of *E. coli*. The expressed protein was affinity purified and then renatured. The immunoreactivity of the expressed recombinant protein was shown by western blot analysis using the specific serum. The experiment was carried out during 2013–14 at Division of Parasitology, Indian Veterinary Research Institute, Izatnagar, U.P., India.

**Results:**

The results of sequencing, restriction digestion analysis, and PCR reaction revealed that cloning of PFR1 gene in pET-32a expression vector and the results of SDS PAGE and Western blot further confirmed its homogeneity and purity. The *in silico* Te-PFR1 (*T. evansi* PFR1) nucleotides sequence analysis revealed its close homology with the other members of the order Kinetoplastida.

**Conclusion:**

We report here the molecular cloning, heterologous expression, and characterization of PFR1, a constituent protein of PFR. Due to its conserved nature, the PFR1 protein could be a prospective vaccine target against multiple *Trypanosoma* species.

## Introduction

Surra, caused by *Trypanosoma evansi*, is prevalent in a wide range of wild and domestic animals including companions in the Indian subcontinent. Surra is a burden to the agriculture-based economy of South Asian countries including India. The disease generally occurs in a chronic form in the large ruminant causing huge loss of production and drought power. The disease is currently managed by chemotherapy using a few drugs available in the market. The emergence of drug resistance and potential toxic nature of the drugs as well as drug residues in animal products have prompted the need for the development of a safe and protective vaccine against the disease (1–4). Paraflagellar rod (PFR) is the constituent proteins of the kinetoplastid flagellum, extending alongside the axoneme from the flagellar pocket to flagellar tip. The PFR is composed of two major proteins PFR1 and PFR2. The slow-migrating protein band in SDS-PAGE gel was defined as PFR1 while the fast migrating band was called PFR2. The nucleotide sequence of PFR is highly conserved throughout the Kinetoplastida and Euglenida (5). Owing to this high sequence homology in trypanosomatids (6), the PFR proteins have the potential of a cross-protective vaccine candidate. We report here the molecular cloning of Te*-*PFR1 gene, its nucleotide sequence comparison, heterologous expression, and characterization.

## Materials and Methods

### In vivo propagation and maintenance of parasites

The whole experiment was conducted during 2013–14 at Division of Parasitology, Indian Veterinary Research Institute (I.V.R.I), Izatnagar, U.P., India. The study was approved by Institutional Animal Ethics Committee of I.V.R.I., No.F.26-1.

A cryostock of *Trypanosoma evansi*, horse isolate was revived and amplified in vivo in Swiss albino mice. At teeming level of parasitemia, heart blood was collected and the trypanosomes were isolated by DEAE-cellulose chromatography (7). The purified parasites were pelleted by centrifugation at 4000 g at 4 °C for 5 min.

### Molecular cloning of PFR1

Total RNA was extracted from the host cell-free trypanosomes using Trizol reagent following the manufacturer’s instructions (Gibco BRL). Double-stranded complementary DNA (cDNA) was synthesized from the total trypanosome RNA using oligo dT primer following the standard protocol (8). Briefly, a 50μl RT-PCR Reaction with RNA was set up with template RNA 15μl (4.5 μg), oligo dT 2μl (100 pM). The mixture was heated at 70 °C for 5 min and snap cooled on ice and the following was added: RT buffer (5X)10μl, dNTPs (10mM) 5 μl, RNase inhibitor (40U/μl) 0.25 μl, *MuMLV* RT (200 U/μl) 2μl, DEPC treated NFW, 15.75 μl and the total volume was made up to 50 μl. The mixture was then incubated at 42 °C for 1h followed by 70 °C for 10 min to inactivate the RT. The entire ORF of *T. evansi* PFR1 gene was amplified from the cDNA by PCR using *Taq* polymerase in a thermal cycler with specific primers (PFR1 forward primer (FPFR): 5’ ATG GCC GCA GTT GAC GAT G 3’ and PFR1 reverse primer (RPFR): 5’ CTA TTC GAG GCG TGC CGG T 3’). The thermal reaction consisted of initial denaturation at 94 °C for 4 min, followed by 32 cycles of denaturation at 94 °C for 45 sec, annealing at 57 °C for 45 sec and extension at 72 °C for 2 min with a final extension at 72 °C for 15 min. The amplified DNA fragment was visualized by electrophoresis in 1% agarose gel containing ethidium bromide and isolated using the QIAquick gel extraction kit (QIAGEN, Germany). The quantification of the purified PCR product was done spectrophotometrically (Nanodrop®, USA). The PCR product was further confirmed by *Hind* III restriction enzyme digestion.

The purified PCR product was ligated to pGEM-T T/A cloning vector following standard protocol (8) and designated as pGEM-T-PFR1. Competent *Escherichia coli* DH5α was transformed with the plasmid construct and were grown on LB agar containing ampicillin at 37 °C overnight. Positive clones were selected and grown overnight in LB media containing ampicillin. The recombinant plasmid was extracted from the transformed DH5α cells (8) and the insert was released by a restriction digestion with *Nco*I/*Pst*I for confirmation. The clones were further confirmed by colony PCR and were custom sequenced for nucleotides.

### Expression of PFR1 gene in pET-32a expression vector

The specific primer-directed PCR amplification of 1770 bp ORF encoding PFR1 was achieved using cDNA as template. The restriction sites for *Eco*RI and *Nco*I enzymes were incorporated into the expression primers. PFR1 forward primer (FEPFR1*Nco*I):5’ ATCACCATGGCCGCAGTTGACGATGCCAC 3’, PFR1 reverse primer (REPFR1*Eco*RI): 5’GCTTGGAATTCCTATTCGAGGCGTGCCG GTGCAG3’. The thermal cycle for PCR amplification of PFR1 ORF was standardized as initial denaturation at 95 °C for 3 min, followed by 30 cycles of denaturation at 94 °C for 30 sec, annealing at 63.6 °C for 50 sec, elongation at 72 °C for 3 min 45 sec and final elongation at 72 °C for 10 min.

The purified PCR product was digested with *Eco*RI and *Nco*I restriction enzymes to sub-clone into the pET32a expression plasmid vector following the standard protocol (8). The recombinant plasmid construct was confirmed by colony PCR and insert release by digestion with *Eco*RI and *Nco*I restriction enzymes.

*E. coli* BL21 (DE3) cells (Promega, USA) were transformed with the recombinant PFR1 plasmid following the standard protocol (8). Five positive colonies were selected from the master plate for induction. The colonies were grown in 5 ml of LB broth overnight at 37 °C with constant shaking at 140 rpm. Ten milliliters of fresh LB broth was added to 100 μl of the overnight grown culture and further incubated at 37 °C until mid-log phase with constant shaking. One milliliter of the culture was collected from each tube as an uninduced control. The rest of the culture was induced by IPTG, added at a final concentration of 1 mM, and incubated at 37 °C with constant shaking. One milliliter of the induced culture was collected at hourly interval starting from 3 h of induction. The cells were pelleted by centrifugation at 13000 rpm and kept at −20 °C till further use.

### Extraction and purification of the recombinant protein

The BL21 cell pellets collected at an hourly interval of induction along with the controls were suspended in 50 μl of SDS-PAGE sample buffer (2X). The sample volume was made up to 100 μl with autoclaved distilled water and then boiled for 10 min in a water bath to lyse the bacterial cells. The lysate was centrifuged at 12000 rpm and 40 μl of the supernatant was analyzed by 12% SDS-PAGE (9) under denaturing conditions at 100V for 2–3 h. The gel was stained with Coomassie Brilliant Blue R-250.

The recombinant PFRI was isolated by pelleting the cells from 100 ml of the induced culture. The cells were resuspended in 5 ml of lysis buffer containing 8M urea and incubated at room temperature for 1–2 h with intermittent vortexing. Subsequently, the debris was pelleted by centrifugation at 13000 rpm for 30 min and the clear supernatant was transferred to a clean tube. Then the supernatant was mixed thoroughly with 800 μl of Ni-NTA aga-rose slurry containing 20 mM imidazole (37.5 μl) and 10 mM β-mercaptoethanol (5 μl) on a rotatory shaker for 1 h. The lysate-resin mixture was loaded onto a 5 ml polypropylene column (Qiagen) and equilibrated with 1X Tris-phosphate buffer (pH 8.0). The flow-through was collected. The column was then washed initially with 10 ml of wash buffer I (pH 6.2) containing 25 mM imidazole (9 μl, pH 7.0) followed by 10 ml of wash buffer II (pH 6.0) containing 30 mM imidazole (9 μl, pH 7.0). Finally, the protein was eluted with 4 ml of elution buffer (pH 4.2) in 500 μl fractions.

### Western blot analysis of the recombinant PFR1 gene

To check the specific reactivity of the purified recombinant PFR1 protein by western blot, about 500 ng of the purified recombinant protein was electrophoresed by SDS-PAGE. The resolved protein was electrotransferred to a nitrocellulose membrane using Tris-Glycine buffer (50 nm Tris base, 380 mM glycine, 0.1% SDS) containing 20% methanol at 100 mA constant current for 3 h. Successful transfer of the protein was confirmed by staining the membrane with Ponceau’s stain. The stain was removed by washing the membrane with TBS buffer. The unbound surface of the membrane was blocked overnight with 3% skimmed milk in TBS, at 4 °C. Following stringent washing with TBS-Tween (0.05%) (3×10min), the membrane was incubated with Ni-NTA anti-histidine HRPase conjugate (Qiagen; 1:1000 dilution) at 37 °C for 1 h. Finally, the membrane was washed thrice with TBS-T and developed with DAB solution (Bangalore Genei, India) in dark. The reaction was stopped by washing the membrane immediately after the color developed. The immune-reactivity of the rPFR1 was further checked against rabbit hyperimmune serum and against reference negative sera.

### Sequence alignment based comparative homology of PFR1

The nucleotide sequences and chromatograms were studied using BioEdit V7.0.5 program (10). The nucleotide sequence of the complete PFR1 CDS was submitted to Gen-Bank (Accession number FJ968743). The edited sequence was translated to ORFs (http://bio.lundberg.gu.se/edu/translat.html). To delineate the functional domains, the translated amino acid sequence was subjected to Simple Modular Architecture Research Tool (SMART) analysis (http://smart.embl-heidelberg.de/#). The translated PFR1 protein sequence was analyzed by ProtParam online program (http://web.expasy.org/protparam/) to examine physicochemical properties like molecular weight, theoretical pI, extinction coefficient, estimated half-life, instability index, aliphatic index, and grand average of hydropathy (GRAVY).

The PFR1 sequences, available in the database from homologous species, were retrieved for BLAST analysis. The homologous nucleotide coding sequences for PFR1 were compared using ClustalW program of MEGA7. The phylogenetic tree was constructed by p-distance matrix using the neighbor-joining method (11) with 1000 bootstrap samplings (12).

## Results

**Fig. 1:**
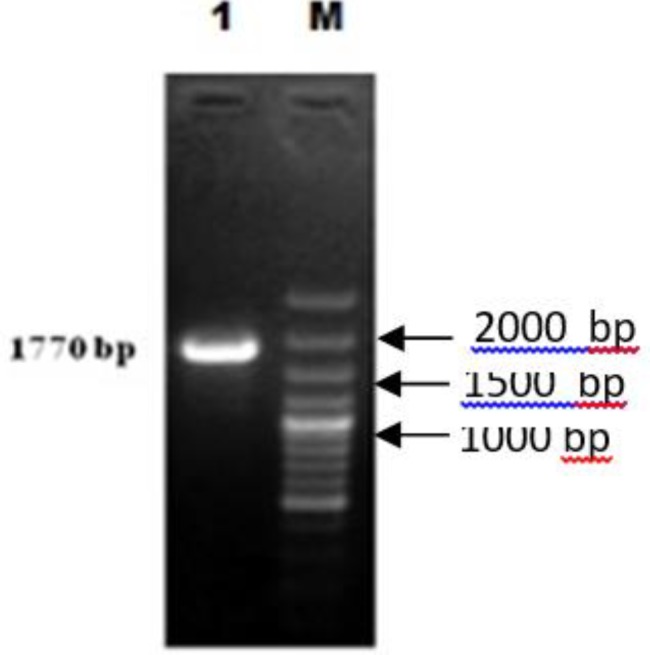
Amplification of 1770 bp PFR1 gene of *T. evansi* Lane M:100 bp plus DNA ladder (Thermo Scientific^TM^ SM0323) / Lane 1: Amplicon of 1770 bp from cDNA template

The entire ORF of *T. evansi* - PFR1 gene (1770 bp) was PCR amplified using specific primers (Fig. 1).

The amplicon was purified from the PCR contaminants using a commercial gel extraction kit (Qiagen, Germany) and the purity was checked by electrophoresis on one percent agarose gel. The molecular integrity of the PCR product was further confirmed by restriction digestion with *Hind* III enzyme which produced two fragments of 1003 bp and 767 bp. The result was consistent as an ORF of *T. evansi* contains a restriction site for *Hind* III at 1003 bp as shown in an *in silico* analysis of a published sequence using NEB cutter analysis tool (http:/nc2.neb.com/NEBcutter2/). The purified 1770 bp DNA fragment was ligated into a T/A cloning vector to facilitate its nucleotide sequencing and characterization. A pGEM-T vector, having an MCS (Multiple cloning sites) incorporated into a *Lac*Z a coding region, was chosen for the easy selection of recombinant clones. Selection of positive *E. coli* DH5α colonies were done by blue-white colony screening method and the white clones were further confirmed for the insert by colony PCR. The positive clone thus selected was custom sequenced for nucleotide.

**Fig. 2:**
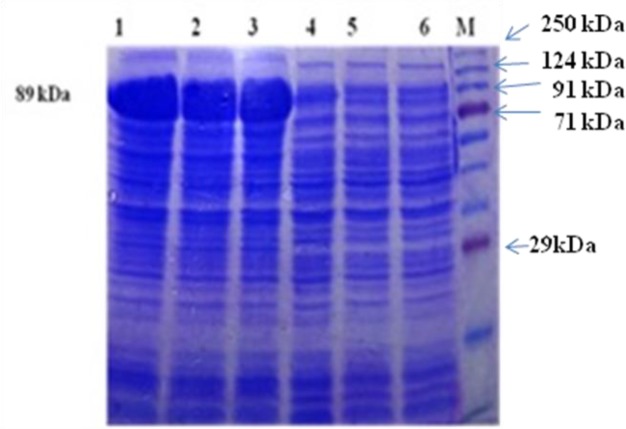
rPFR1 protein production by rec.BL21 DE3 *E.coli* cells by SDS-PAGE Lane M: Prestained protein ladder (Puregene, Genetix, PG-PMT 2922) Lane 1: 4h post induction Lane 2: 6h post induction Lane 3: 8h post induction Lanes 4–6: Uninduced control

The transformed BL-21 cells were grown in a medium containing the standard inhibitory concentrations of ampicillin (100μg/ml). The recombinant clones were checked by colony PCR and insert release analysis by restriction digestion with *EcoR*I and *Nco*I. A high-level expression of PFR1 protein was noted after six hours of induction with 1mM IPTG. The molecular mass of the expressed fusion protein was determined as 89 kDa by SDS-PAGE (Fig. 2).

**Fig. 3:**
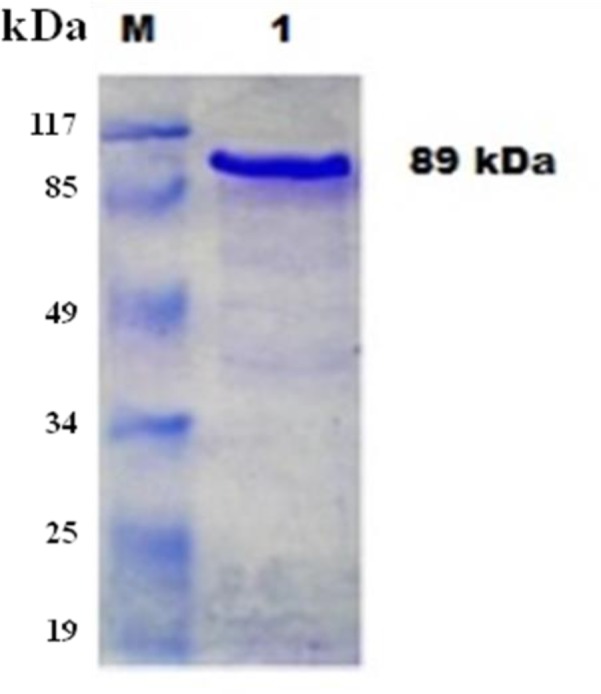
Purified rPFR1 protein by Ni-NTA affinity chromatography /Lane M: Prestained molecular, weight protein marker (MBI Fermentas, SM0441) Lane 1: Elute 1 from NI-NTA agarose column

**Fig. 4:**
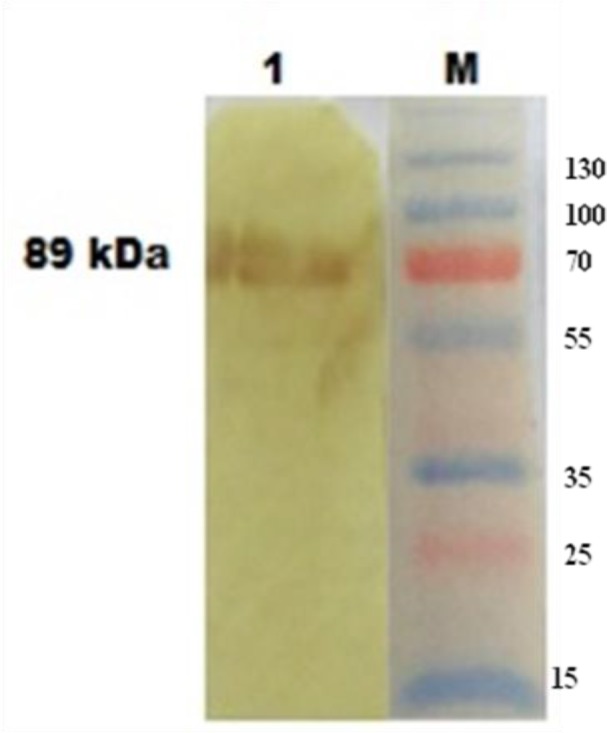
The immunoreactivity of rPFR1 protein by western blot analysis with rabbit hyper immune serum raised against native whole cell lysates of *T. evansi* Lane M: Pre-stained protein molecular weight marker (Thermofisher Scientific, 26619) Lane 1: Purified PFR1 protein

The recombinant Te-PFR1 protein was purified by affinity chromatography using Ni-NTA agarose beads, and was eluted by imidazole mediated competitive recovery at low pH (Fig. 3). The refolding of the eluted protein was achieved by dialysis against decreasing concentration of urea at 6 h interval and finally against PBS pH 7.4 for 24 h at 4 °C. The heterologous expression of the recombinant protein was confirmed by immunoblot analysis using specific Ni-NTA HRP conjugate. Further, an immunoblot analysis with hyperimmune sera confirmed the immunore-activity of the expressed recombinant protein (Fig. 4).

The molecular conformation of the recombinant Te-PFR1 was studied *in silico*. The SMART analysis of the deduced amino acid sequence revealed four coiled regions spanning 124–199, 249–278, 313–339 and 477–517 amino acid (aa) residues and one low complexity region at 342–360 aa residues. The ProtParam program predicted the isoelectric point (pI) of Te-PFR1 protein as 5.77, which indicates its slight positive charge and an instability index of 53.55 was suggestive of its unstable nature. The GRAVY index of −0.825 was indicative of a hydrophobic and insoluble nature of Te-PFR1 protein (13).

The BLAST analysis retrieved similar sequences of PFR1 from the public database. Fourteen highly homologous sequences PFR1 *viz. Trypanosoma evansi* Bikaner (Indian) isolate (JQ909241), *Trypanosoma evansi* China isolate (EU366960), *Trypanosoma brucei gambiense* (XM_011774118), *Trypanosoma brucei* (XM_838928), *Trypanosoma grayi* (XM_009309683), *Trypanosoma cruzi* (AF005195), *Leishmania donovani* (XM_003862559), *Leishmania infantum* (XM_003392645), *Leishmania panamensis* (XM_010702480), *Leishmania braziliensis* (XM_001566967), *Leishmania major* (XM_003722209), *Leptomonas pyrrhocoris* (XM_015802553), *Leishmania mexicana* (AY198411) and *Crithidia deanei* (AY785777) were selected for comparison. The PFR1 gene sequences were aligned, the sequence distance was estimated and the neighbour-joining phylogenetic tree was constructed.

The model (Tamura-Nei with discrete gamma distribution *i.e.* TN93+G) with lowest BIC score (13183.401) was considered to be best model in MEGA7 for describing the substitution pattern.

The sequence identity (Table 1) of *T. evansi* Izatnagar isolate was maximum with the *T. evansi* Bikaner (Indian) isolate (100%) and *T. evansi* China isolate (99.9%).

**Table 1:** The PFR1 sequence of *Trypanosoma evansi* Izatnagar isolate was compared to the same from some other members of Kinetoplastida available in the GenBank. The upper diagonal values represent sequence distance, whereas the lower diagonal values represent standard error

***SPP.***	***Sequence distance and standard error***															
*Trypanosoma evansi*															
Izatnagar (Indian)															
isolate (FJ968743)		0.000	0.001	0.000	0.001	0.016	0.019	0.016	0.016	0.016	0.016	0.016	0.016	0.016	0.026
*Trypanosoma evansi*															
Bikaner (Indian)															
isolate (JQ909241)	0.000		0.001	0.000	0.001	0.016	0.019	0.016	0.016	0.016	0.016	0.016	0.016	0.016	0.026
*Trypanosoma evansi*															
China isolate															
(EU366960)	0.001	0.001		0.001	0.001	0.016	0.019	0.016	0.016	0.016	0.016	0.016	0.017	0.017	0.026
*Trypanosoma brucei gambiense*															
(XM_011774118)	0.000	0.000	0.001		0.001	0.016	0.019	0.016	0.016	0.016	0.016	0.016	0.016	0.016	0.026
*Trypanosoma brucei*															
(XM_838928)	0.002	0.002	0.002	0.002		0.016	0.019	0.016	0.016	0.016	0.016	0.016	0.017	0.016	0.026
*Trypanosoma grayi*															
(XM_009309683)	0.195	0.195	0.196	0.195	0.196		0.013	0.014	0.014	0.014	0.014	0.013	0.012	0.014	0.024
*Trypanosoma cruzi*															
(AF005195)	0.205	0.205	0.206	0.205	0.206	0.150		0.015	0.016	0.015	0.015	0.016	0.015	0.015	0.023
*Leishmania donovani*															
(XM_003862559)	0.215	0.215	0.216	0.215	0.215	0.164	0.176		0.001	0.005	0.006	0.003	0.006	0.003	0.023
*Leishmania infantum*															
(XM_003392645)	0.215	0.215	0.216	0.215	0.215	0.166	0.176	0.001		0.005	0.006	0.003	0.006	0.003	0.023
*Leishmania panamensis*															
(XM_010702480)	0.215	0.215	0.216	0.215	0.215	0.170	0.175	0.038	0.039		0.002	0.005	0.006	0.005	0.022
*Leishmania braziliensis*															
(XM_001566967)	0.215	0.215	0.216	0.215	0.215	0.170	0.175	0.040	0.041	0.005		0.006	0.007	0.005	0.022
*Leishmania major*															
(XM_003722209)	0.218	0.218	0.219	0.218	0.218	0.164	0.182	0.010	0.011	0.040	0.042		0.006	0.004	0.022
*Leptomonas pyrrhocoris*															
(XM_015802553)	0.219	0.219	0.220	0.219	0.219	0.154	0.171	0.050	0.052	0.055	0.057	0.052		0.006	0.022
*Leishmania mexicana* (AY198411)	0.221	0.221	0.221	0.221	0.221	0.167	0.177	0.016	0.017	0.038	0.040	0.019	0.054		0.024
*Crithidia deanei*															
(AY785777)	0.339	0.339	0.341	0.339	0.342	0.329	0.318	0.307	0.310	0.298	0.295	0.304	0.279	0.315	

**Fig. 5:**
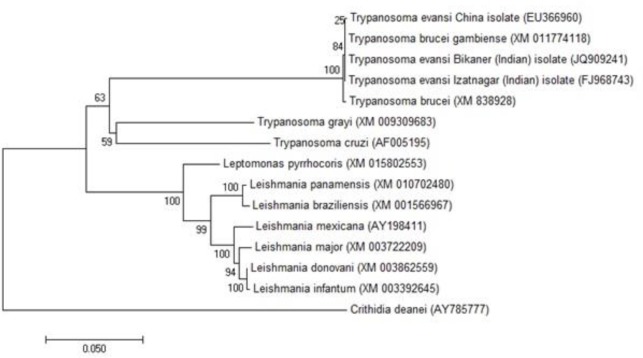
Phylogenetic tree constructed based on PFR1 nucleotide sequence using the Tamura-Nei model with uniform Gamma distribution and 1000 bootstrap multiplication

The *T. brucei gambiense* and *T. brucei* had a sequence identity of 100% and 99.9% with *T. evansi* Izatnagar isolate (Fig. 5). Further, a close sequence homology of *Te*-PFR1 was found when aligned against that of *L. panamensis, L. braziliensis, L. donovani, L. major, L. infantum, T. grayi, L. pyrrhocoris, L. Mexicana, T. cruzi and C. deanei* (Table 1).

## Discussion

Surra is one of the most important vector borne haemoprotozoan diseases of tropical and subtropical countries. Importance of trypanosomosis ranks high due to its devastating effects on the livestock health and productivity leading to severe economic losses to the dairy industry (4, 14). Presently the control measures rely solely on chemotherapy.

There are only a few drugs available in the market with low therapeutic index and high toxicity. Presence of drug residues in the animal products and emergence of drug-resistant strains necessitate development of alternative control measures including a sustainable protective vaccine. Development of a protective vaccine is still a distant dream owing to the antigenic variation displayed by the organism. Since VSG no longer remains an impressive choice as a vaccine candidate, emphasis is laid on testing of non-variable vaccine targets for their immunoprotective potentials (3) like PFR1 owing to its strategic location and conserved nature among different species (15).

In the present communication, we report the molecular cloning of Te*-*PFR1 gene, its nucleotide sequence comparison, heterologous expression, and characterization. The principal finding of this study was the identification of the PFR1 gene in *T. evansi* of Indian isolate by sequencing the recombinant plasmid pGEMT-PFR1 three times in both directions with no changes. A search for sequences relating to the PFR1 gene of *T. evansi* in the GenBank at the National Center for Biotechnology Information revealed several PFR1 nucleotide sequences. The search resulted in the identification of complete DNA sequences of PFR1 genes that were homologous to the newly reported Te*-* PFR1 gene.

This is the first information on the sequence generated from an Indian isolate of *T. evansi* and submitted to GenBank from India (Accession No. FJ968743). The amplicon of PFR1 gene (1770 bp) is 30 nucleotides less than that of PFR2 gene (1800 bp). In vitro expression of PFR1 protein was carried out using pET32a vector in BL21 DE3 strain of *E. coli,* as His-tagged fusion protein incorporating *Eco*RI and *Nco*I restriction sites in the expression primers since the sequence information of PFR1 gene revealed the absence of the sites for either of these restriction enzymes. A high level of expression PFR1 was noted following six hours of induction of the culture with 1mM IPTG. pET 31a vector was used and expressed PFR1 of *T. evansi* in BL21 DE3 strain of *E.coli*. The workers reported maximum yield of the protein at 6 h post-induction. The expressed protein was purified chromatographically using Ni-NTA agarose beads under denaturing conditions to lyse and recover the cytoplasmic contents into the lysis buffer supernatant and was renatured by dialysis against tris-saline (pH 7.4). Western blot analysis of expressed histidine-tagged recombinant protein confirmed their identity. When incubated with hyperimmune serum, the immunoreactivity at the unique 89 kDa region specific for the PFR1 on the nitro-cellulose membrane confirmed the presence and purity of the recombinant protein. The mature PFR1 protein is comprised of 590 amino acids with a deduced molecular weight of 73 kDa (16). In the present investigation, since the pET32a vector contains HisTag, Trx-Tag and S-Tag, about 16 kDa molecular mass was added to the recombinant PFR1 protein. The expressed fusion protein was resolved as 89 kDa molecular mass by SDS-PAGE.

Earlier studies reported in vitro expression of PFR2 protein using pET32a vector, as His tagged fusion protein incorporating *EcoR*I and *Hind*III restriction sites in the expression primers. The mature PFR2 protein is composed of 600 aminoacids (4). The expressed fusion protein was resolved at the Molecular weight of 88 kDa in SDS-PAGE. The workers reported maximum yield of protein 6h post induction. We got similar findings with rPFR1. Both crude and recombinant version of PFR protein from kinetoplastids showed strong immunogenicity in rodent models (17–19). Immunological relatedness between salivarian and stercorarian groups of trypanosomes is rarely investigated. Recently, *T. evansi* crude antigen showed cross reactivity with *T. cruzi* positive sera obtained from chagasic patients (20), signifying the presence of common antigens, such as PFR related proteins between these two trypanosome species (16). Moreover, it is interesting to note that the PFR proteins bear no homology to any human and livestock protein (21).

## Conclusion

The close homology in nucleotide sequence of PFR1 gene of kinetoplastids denotes its conserved nature. The findings give credence to the hypothesis that the PFR molecule(s) as a vaccine candidate has potential to induce protective immunity against many other species of *Trypanosoma* crossing the strain barrier.

## References

[1] Borst P Rudenko G. Antigenic variation in African trypanosomes. Science. 1994; 264(5167):1872–3. 7516579 10.1126/science.7516579

[2] Singh V Singh A Chhabra MB. Polypeptide profiles and antigenic characterization of cell membrane and flagellar preparations of different stocks of *Trypanosoma evansi*. Vet Parasitol. 1995; 56(4):269–79. 7754604 10.1016/0304-4017(94)00690-e

[3] Donelson JE Kent HK EL-sayed MN. Multiple mechanisms of immune evasion by African trypanosomes. Mol Biochem Parasitol. 1998; 91: 51–56. 9574925 10.1016/s0166-6851(97)00209-0

[4] Maharana BR Tewari AK Singh V. An overview on kinetoplastid paraflagellar rod. J Parasit Dis. 2015; 39(4):589–95. 26688619 10.1007/s12639-014-0422-x PMC4675581

[5] Portman N Gull K. The Paraflagellar rod of Kinetoplastid Parasites: From Structures to components and function. Int J Parasitol. 2010; 40(2):135–48. 19879876 10.1016/j.ijpara.2009.10.005 PMC2813431

[6] Maga JA Sherwin T Francis S Genetic dissection of the *Leishmania* paraflagellar rod, a unique flagellar cytoskeleton structure. J Cell Sci. 1999; 112 (Pt 16):2753–63. 10413682 10.1242/jcs.112.16.2753

[7] Lanham SH Godfrey DG. Isolation of salivarian trypanosomes from man and other animals using DEAE-cellulose. Exp Parasitol. 1970; 28(3):521–34. 4993889 10.1016/0014-4894(70)90120-7

[8] Sambrook J Russel DW. Molecular cloning: A laboratory manual 3rd ed Cold spring Harbor Laboratory Press, Cold spring Harbor, NY 2001.

[9] Laemmli UK. Cleavage of structural proteins during assembly of the head of bacteriophage T4. Nature. 1970; 227(5259):680–5. 5432063 10.1038/227680a0

[10] Hall T. BioEdit: important software for molecular biology. GERF Bul Bio. 2011; 2:60–61.

[11] Saitou N Nei M. The neighbour-joining method: a new method for reconstructing phylogenetic trees. Mol Bio Evol. 1987; 4(4):406–25. 3447015 10.1093/oxfordjournals.molbev.a040454

[12] Kumar S Stecher G Tamura K. MEGA7: Molecular Evolutionary Genetics Analysis version 7.0 for bigger datasets. Molecular Biology and Evolution. Mol Biol Evol. 2016; 33(7):1870–4. 27004904 10.1093/molbev/msw054 PMC8210823

[13] Gasteiger E Hoogland C Gattiker A Protein Identification and Analysis Tools on the ExPASy Server; (In) Walker John M. (ed): The Proteomics Protocols Handbook, Humana Press 2005; 571–607.

[14] Yang C Suo X Huang X Protection of mice against homologous or heterologous infections with antiserum mixture to the predominant variable antigen type repertoire of *Trypanosoma evansi* YNB stock. Exp Parasitol. 2007; 116(1):53–8. 17223107 10.1016/j.exppara.2006.11.010

[15] Maharana BR Rao JR Tewari AK Molecular characterization of Para Flagellar Rod Protein Gene (PFR) of in *Trypanosoma evansi*. J Appl Anim Res. 2014; 1: 1–5.

[16] Abdille MH Li SY Jia Y Evidence for the existence of paraflagellar rod protein 2 gene in *Trypanosoma evansi* and its conservation among other kinetoplastid parasites. Exp Parasitol. 2008; 118(4):614–8. 18179792 10.1016/j.exppara.2007.11.011

[17] Luhrs KA Fouts DL Manning JE. Immunization with recombinant paraflagellar rod protein induces protective immunity against *Trypanosoma cruzi* infection. Vaccine. 2003; 21(21–22):3058–69. 12798650 10.1016/s0264-410x(03)00108-7

[18] Saravia NG Hazbón MH Osorio Y Protective immunogenicity of the Paraflagellar rod protein 2 of *Leishmania mexicana*. Vaccine. 2005; 23(8):984–95. 15620471 10.1016/j.vaccine.2004.07.044

[19] Wrightsman RA Miller MJ Saborio JL Manning JE. Pure paraflagellar rod protein protects mice against *T.cruzi* infection. Infect Immun. 1995; 63(1):122–5. 7806347 10.1128/iai.63.1.122-125.1995 PMC172967

[20] Desquesnes M Bosseno MF Brenière SF. Detection of chagas infections using *Trypanosoma brucei* crude antigen demonstrates high cross-reactions with *Trypanosoma cruzi*. Infect Genet Evol. 2007; 7(4):457–62. 17337255 10.1016/j.meegid.2007.01.007

[21] Clark AK Kovtunovych G Kandlikar S Cloning and expression analysis of two novel paraflagellar rod domain genes in *Trypanosoma cruzi*. Parasitol Res. 2005; 96(5):312–20. 15918067 10.1007/s00436-005-1370-2

